# Caspase-3-like activity determines the type of cell death following ionizing radiation in MOLT-4 human leukaemia cells

**DOI:** 10.1054/bjoc.2000.1322

**Published:** 2000-08-16

**Authors:** D Coelho, V Holl, D Weltin, T Lacornerie, P Magnenet, P Dufour, P Bischoff

**Affiliations:** Laboratoire de Cancérologie Expérimentale et de Radiobiologie, IRCAD, Hôpitaux Universitaires, BP 426, Strasbourg Cedex, F-67091, France; Centre Paul Strauss, Strasbourg, F-67000, France

**Keywords:** apoptosis, necrosis, caspase, ionizing radiation, MOLT-4

## Abstract

Caspases, a family of cysteine proteases, play a central role in the pathways leading to apoptosis. Recently, it has been reported that a broad spectrum inhibitor of caspases, the tripeptide Z-VAD-fmk, induced a switch from apoptosis to necrosis in dexamethasone-treated B lymphocytes and thymocytes. As such a cell death conversion could increase the efficiency of radiation therapy and in order to identify the caspases involved in this cell death transition, we investigated the effects of caspase-3-related proteases inhibition in irradiated MOLT-4 cells. Cells were pretreated with Ac-DEVD-CHO, an inhibitor of caspase-3-like activity, and submitted to X-rays at doses ranging from 1 to 4 Gy. Our results show that the inhibition of caspase-3-like activity prevents completely the appearance of the classical hallmarks of apoptosis such as internucleosomal DNA fragmentation or hypodiploid particles formation and partially the externalization of phosphatidylserine. However, this was not accompanied by any persistent increase in cell survival. Instead, irradiated cells treated by this inhibitor exhibited characteristics of a necrotic cell death. Therefore, functional caspase-3-subfamily not only appears as key proteases in the execution of the apoptotic process, but their activity may also influence the type of cell death following an exposure to ionizing radiation. © 2000 Cancer Research Campaign


*British Journal of Cancer* (2000) **83**(5), 642–649
© 2000 Cancer Research Campaign
doi: 10.1054/ bjoc.2000.1322, available online at http://www.idealibrary.com on IDEAL®

### Caspase-3-like activity determines the type of cell death following ionizing radiation in MOLT-4 human leukaemia cells

**D Coelho[1], V Holl[1], D Weltin[1], T Lacornerie[2], P Magnenet[2], P Dufour[1] and P Bischoff[1]**

[1]Laboratoire de Cancérologie Expérimentale et de Radiobiologie, IRCAD, Hôpitaux Universitaires, BP 426, F-67091 Strasbourg Cedex, France; [2]Centre Paul Strauss, F-67000 Strasbourg, France

**Summary** Caspases, a family of cysteine proteases, play a central role in the pathways leading to apoptosis. Recently, it has been reported that a broad spectrum inhibitor of caspases, the tripeptide Z-VAD-fmk, induced a switch from apoptosis to necrosis in dexamethasone-treated B lymphocytes and thymocytes. As such a cell death conversion could increase the efficiency of radiation therapy and in order to identify the caspases involved in this cell death transition, we investigated the effects of caspase-3-related proteases inhibition in irradiated MOLT-4 cells. Cells were pretreated with Ac-DEVD-CHO, an inhibitor of caspase-3-like activity, and submitted to X-rays at doses ranging from 1 to 4 Gy. Our results show that the inhibition of caspase-3-like activity prevents completely the appearance of the classical hallmarks of apoptosis such as internucleosomal DNA fragmentation or hypodiploid particles formation and partially the externalization of phosphatidylserine. However, this was not accompanied by any persistent increase in cell survival. Instead, irradiated cells treated by this inhibitor exhibited characteristics of a necrotic cell death. Therefore, functional caspase-3-subfamily not only appears as key proteases in the execution of the apoptotic process, but their activity may also influence the type of cell death following an exposure to ionizing radiation. © 2000 Cancer Research Campaign

**Keywords:** apoptosis; necrosis; caspase; ionizing radiation; MOLT-4

To date, radiotherapy remains one of the most effective anticancer treatments. Its efficiency relies on the physical targeting of the tumour mass and for some types of lymphomas on the induction of apoptosis into the malignant cells by ionizing radiation (IR) (Zhivotovsky et al, 1999). However, despite the steadily increasing knowledge in our understanding of apoptosis, its triggering by IR still remains elusive and could involve DNA damage, membrane alterations, ceramide formation (Haimovitz-Friedman et al, 1994), reactive oxygenated species generation (Buttke and Sandstrom, 1994), up-regulation of CD95/Fas/APO-1 (Belka et al, 1998) and caspases activation (Hallan et al, 1997), including caspase-3 (Datta et al, 1997). In the last few years, it has become clear that the caspases network is a central actor of the apoptotic machinery. Indeed, these cysteine proteases are involved in the early phases of apoptosis: induction and transduction of the death signal, as well as in the final step: the execution phase. The number and the functional importance of their substrates in the cell cycle regulation, DNA repair, cytoskeleton and nuclear lamina suggest that caspase activation leads to cell demise (Cohen, 1997). Thus, modifying the activity of one of these proteases could constitute a fruitful approach for the modulation of the apoptotic process and possibly other types of cell death (Mignon et al, 1998; Thornberry and Lazebnik, 1998). While in several studies it has been reported that in fact, caspase inhibition prevents apoptotic hallmarks, some recent works also showed that the inhibition of caspases does not affect cell survival but shifts cell death from apoptosis to necrosis, suggesting the existence of a common initial pathway between these two forms of cell death (Hirsch et al, 1997; Lemaire et al, 1997).

Although necrosis and the subsequent inflammation should be avoided in morphogenesis, immune regulation or tissue homeostasis, its occurrence might be of interest in anticancer treatment since it could enhance the recruitment of macrophages and T cells which in turn could participate to the eradication of tumour cells (Reiter et al, 1999). Necrotic cell death was also found to activate and mature dendritic cells (Sauter et al, 2000). Moreover, necrotic cells, by spreading out their intracellular content including active enzymes and reactive oxygenated species, could also damage surrounding cells.

Recently, characterization of the cleavage site of some caspase substrates gave rise to specific oligopeptide inhibitors, such as Ac-DEVD-CHO, a competitive and reversible inhibitor of caspase-3-like enzymes (Nicholson et al, 1995). By using this tetrapeptide, we have investigated whether the inhibition of caspase-3-like activity could influence the morphological and biochemical features of radiation-induced apoptosis in the human leukaemic cell line MOLT-4. Cells were pretreated with Ac-DEVD-CHO (300 μM) and submitted to X-rays at doses ranging from 1 y to 4 Gy. Our results show that the inhibition of caspase-3-like proteases prevented the appearance of hallmarks of apoptosis such as internucleosomal DNA fragmentation or hypodiploid particle formation. Phosphatidylserine externalization was partially inhibited by Ac-DEVD-CHO, suggesting that caspase-3-like enzymes are involved in this apoptotic feature. Although inhibition of DEVD-specific proteases could prevent apoptosis, no persistent increase in cell survival nor clonogenic potential was recorded. Moreover, irradiated cells treated by this caspase-3 inhibitor exhibited characteristics

*Received 14 October 1999*
*Revised 2 May 2000*
*Accepted 7 May 2000*

*Correspondence to:* P Bischoff

The two first authors contributed equally to this study.

of a necrotic cell death. These results not only indicate that caspase-3 is a central protease in the execution of the apoptotic process, but that its inhibition could also orient cell death toward necrosis.

### MATERIALS AND METHODS

### Chemicals

Trypan blue, EDTA, EGTA, Tris, DTT, CHAPS, HEPES, NaCl, $MgCl_2$ and propidium iodide were purchased from Sigma Chemical Co (St Louis, MO). The inhibitor Ac-DEVD-CHO and the colorimetric substrate Ac-DEVD-pNA of caspase-3-related proteases were obtained from Neosystem (Strasbourg, France). Leupeptin, pepstatin, aprotinin, PMSF, bovine serum albumin (BSA) and RNase A, DNase free were purchased from Boehringer Mannheim Corp (Indianapolis, IN). Triton X-100 and Tween 20 were purchased from Calbiochem-Novabiochem Corp (San Diego, CA).

### Cell culture

Cells were grown in suspension in RPMI 1640-Glutamax (Life Technologies, Inc, Gaithersburg, MD) supplemented with 10% heat inactivated fetal calf serum (DAP, Vogelgrün, France), 1 mM sodium pyruvate, 1 mM non-essential aminoacids and 50 µg/ml gentamycin (Life Technologies, Inc). They were maintained at 37°C in a humidified atmosphere containing 5% $CO_2$. Cells were harvested during exponential growth and mortality never exceeded 5% as assessed by Trypan blue exclusion test.

### Irradiation procedure

Cells in exponential phase of growth were harvested by centrifugation and brought to $10^6$ cells/ml in fresh medium. Cells were irradiated at room temperature by an X-ray linear accelerator of 15 MV (Centre Paul Strauss, Strasbourg, France) at a dose rate of approximately 2.50 Gy/min.

### DNA labelling and flow cytometry analysis

Hypodiploid DNA was measured as described in previous work (Nicoletti et al, 1991). Briefly, $10^6$ cells were centrifuged and fixed in 1 ml cold 70% ethanol at 4°C for one hour, washed once in PBS (Life Technologies), EDTA 2 mM, and resuspended in 1 ml of PBS containing 0.25 mg Rnase A, EDTA 2 mM and 0.1 mg of propidium iodide. After incubation in the dark at 37°C for 30 min, cells were kept at 4°C for less than 12 hours until analysis. The fluorescence of 10 000 cells was analysed using a flow cytometer (FACScan, Becton Dickinson, San Jose, CA).

### Annexin V-FITC and propidium iodide simultaneous staining

Phosphatidylserine externalization was assessed by measuring annexin V-FITC binding using a kit from Euromedex (Souffelweyersheim, France) and according to the manufacturer's instructions. Briefly, cells were washed in cold RPMI and for each sample $5 \times 10^5$ cells were resuspended in 100 µl of the reaction buffer (10 µl of binding buffer 10 ×, 10 µl of propidium iodide, 1 µl of annexin V-FITC and 79 µl of deionized water). After 15 min of incubation in the dark at room temperature, each reaction was diluted with binding buffer 1 × to obtain a final volume appropriate for flow cytometry (at least 200 µl). Samples were then immediately analysed using a flow cytometer (Becton Dickinson).

### Western blot

$2 \times 10^6$ cells were lysed for 20 min at 4°C in lysis-buffer (Tris HCl 50 mM pH 7.5, MgCl2 8 mM, EDTA 5 mM, EGTA 0.5 mM, leupeptin 10 µg/ml, pepstatin 10 µg/ml, aprotinin 10 µg/ml, PMSF 1 mM, NaCl 250 mM, Triton X-100 1%). Protein content was assessed using BCA protein assay (Pierce, Rockford, IL). Equal amounts of protein were submitted to a 12% SDS-polyacrylamide gel electrophoresis at 100 V for 90 min. Proteins were then electrophoretically transferred onto a nitrocellulose membrane (Pall Gelman Sciences, Ann Arbor, MI). Membranes were incubated in blocking buffer (Tris HCl 7.5 pH 10 mM, NaCl 100 mM, Tween 20 0.1%, bovine serum albumin 3%) for 60 min at 37°C, and then incubated with mouse monoclonal antibody antihuman CPP32/Casp-3, mouse monoclonal antibody antihuman Casp-7 (1/10 000 dilution, Transduction Laboratories, Lexington, KY) or mouse anti-actin monoclonal antibody (Chemicon International, Inc, Temecula, CA). Membranes were washed twice (Tris HCl 7.5 pH 10 mM, NaCl 100 mM, Tween 20 0.1%), reblocked in BSA-containing buffer, incubated with peroxydase-conjugated goat anti-mouse antibody (1/50 000 dilution, Jackson ImmunoResearch Laboratories, Inc, West Grove, PA) and washed twice in TBS buffer. Immunoblots were visualized by Opti 4CN (Bio-Rad Laboratories, Hercules, CA). Intensity of bands was measured using NIH Image software.

### Catalytic activity

$2 \times 10^7$ cells were washed twice in PBS containing EDTA 2 mM and incubated in lysis buffer (HEPES 50 mM, pH 7.4, CHAPS 0,1%, DTT 1 mM, EDTA 0.1 mM) on ice for 20 min. Lysates were centrifuged at 10 000 g at 4°C during 10 min and supernatants were stored at –70°C until analysis. 20 µl of each supernatant were then incubated with 10 µl of Ac-DEVD-pNA (200 µM) and 70 µl of assay buffer (HEPES 50 mM, pH 7.4, NaCl 100 mM, CHAPS 0,1%, DTT 10 mM, EDTA 1 mM, glycerol 10%) at 37°C during 1 hour. Samples were read at 405 nm.

### TUNEL assay

$10^7$ cells were washed once in PBS and fixed in a 4% paraformaldehyde solution at 4°C during 20 min. Cells were then washed in PBS, incubated 20 min at 4°C in a 70% methanol solution containing 0.3% $H_2O_2$, washed in PBS, incubated 2 min at 4°C in 2% Triton, washed in PBS, incubated 80 min at 37°C in a terminal deoxynucleotidyl transferase working buffer (Boehringer Mannheim), washed in PBS and analysed by flow cytometry.

### LDH release

Cell lysis was assessed by measuring LDH release using a Cytotoxicity Detection kit from Boehringer Mannheim according to the manufacturer's instructions. Samples were read at 490 nm on a microplate MRX reader (Dynex Technologies, Issy-les-Moulineaux, France) and results were expressed as follows: 100 × LDH in supernatants (cytoplasmic LDH+LDH in supernatants).

*© 2000 Cancer Research Campaign*

### Clonogenic potential assay

After X-irradiation, cells were washed with RPMI 1640 and seeded in triplicate in a petri-dish at a concentration of 300 cells/dish in a plasma medium containing human AB serum 20% (Etablissement de Transfusion Sanguine de Strasbourg, France), BSA 10%, RPMI 1640 50%, plasma bovine citrate 10% (Life Technologies), $CaCl_2$ and asparagine 10%, and were allowed to grow for 14 days at 37°C in a humidified atmosphere containing 5% $CO_2$. Cell-forming colonies number was determined at each X-ray dose.

### Alamar blue proliferation test

This assay was performed according to manufacturer's instructions (Alamar, Sacramento, CA). Briefly, after X-irradiation cells were seeded at 5000 cells/well (200 μl) in a 96-wells microplate. Each experimental assay was performed in triplicate. After 4 days of incubation, supernatants were replaced with fresh culture medium and 20 μl of Alamar Blue working solution was added to each well. After incubation at 37°C in a humidified atmosphere containing 5% $CO_2$, during 4 hours, plates were read at 590 nm (excitation: 560 nm) on a microplate Flow Multiscan reader (Dynex Technologies).

### RESULTS

### Irradiation of MOLT-4 cells induces caspase-3-like activity blocked by Ac-DEVD-CHO

We first examined the involvement of caspase-3-like activity in both spontaneous and X-rays-induced apoptosis using Ac-DEVD-pNA, a colorimetric substrate that mimics the cleavage site of caspase-3-like proteases endogenous substrate PARP (Gurtu et al, 1997). MOLT-4 cells were pretreated with a reversible and potent inhibitor of the caspase-3-like activity, Ac-DEVD-CHO (300 μM), 1 hour prior to 2 Gy irradiation. Ac-DEVD-pNA cleaving activity was then measured after 6, 12 and 24 hours of culture. This colorimetric assay shows that a pronounced increase of the DEVD-specific activity occurs 12 hours after irradiation in untreated MOLT-4 cells (Figure 1A) and culminates at 24 hours. In the presence of the inhibitor, no significant DEVDase activity was recorded throughout the culture time. Our results confirm that caspase-3-like proteases are activated following ionizing radiation (Datta et al, 1997; Hallan et al, 1997; Machleidt et al, 1998).

### Caspase-3-like activity induced by irradiation involves caspase-3 and caspase-7

The proteolytic activity detected using Ac-DEVD-pNA could be due to caspase-3 but also to another protease such as caspase-7 (Thornberry et al, 1997). These cysteine proteases are expressed in cells as inactive zymogens and converted during the apoptotic process in their active forms by proteolytic processing after a specific aspartate residue. Inactive caspase-3 is a 32 kDa proform

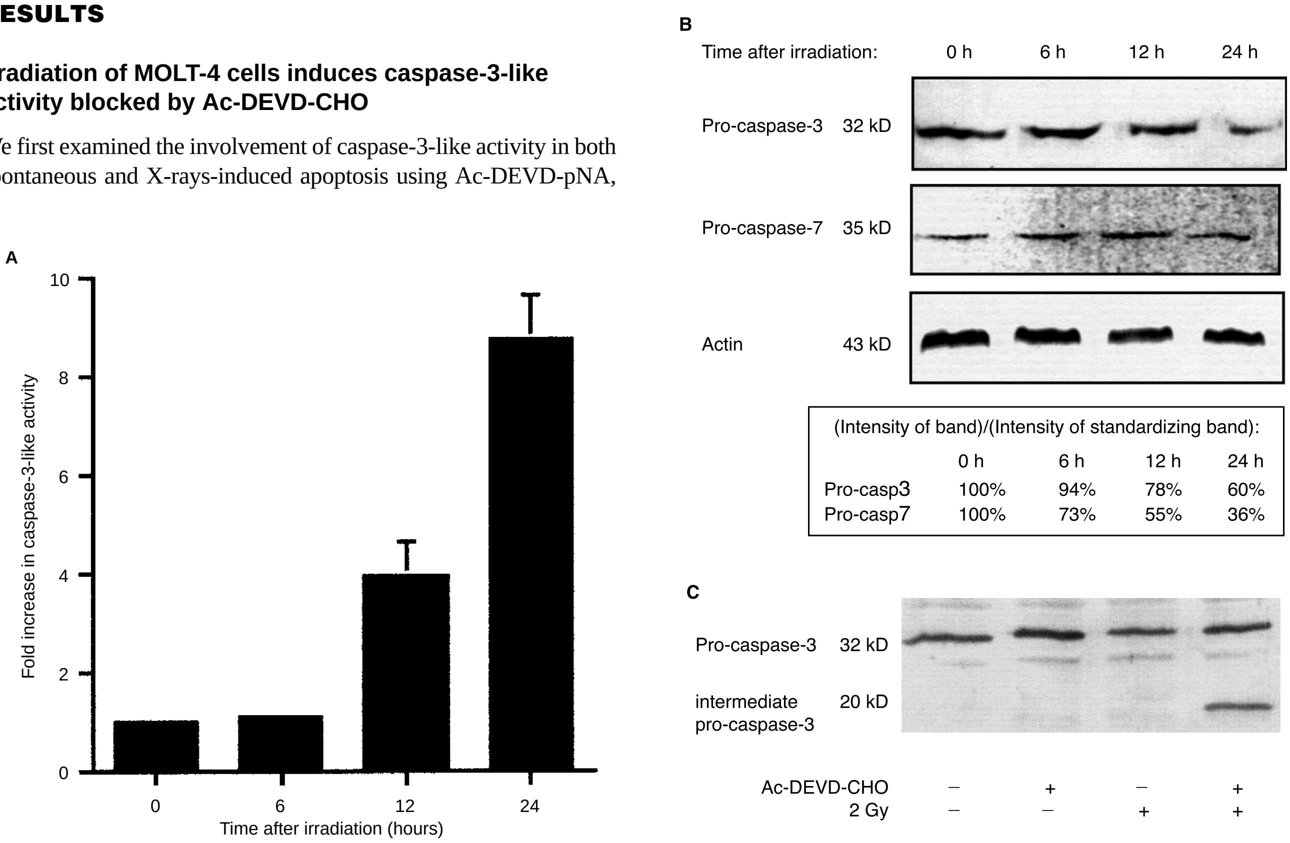

| (Intensity of band)/(Intensity of standardizing band): | 0 h | 6 h | 12 h | 24 h |
|---|---|---|---|---|
| Pro-casp3 | 100% | 94% | 78% | 60% |
| Pro-casp7 | 100% | 73% | 55% | 36% |

**Figure 1** Activation of caspase-3-like proteases by ionizing radiation. (**A**) MOLT-4 cells were cultured for 24 hours following 2 Gy X-irradiation. At the indicated times, cells were washed, lysed and hydrolysis of Ac-DEVD-pNA was measured at 405 nm as described in Materials and Methods. The fold increase in DEVD-pNA cleaving activity was calculated by comparing the percentage of enzymatic activity in control cells versus the percentage of enzymatic activity in cells treated with Ac-DEVD-CHO. Each point represents a mean of three independent experiments in duplicate ± SD. (**B**) Western blot analysis of caspase-3, caspase-7 and actin. At the indicated times, 50 μg of proteins per lane, isolated from cell lysates were submitted to 12% SDS-PAGE. Values of intensity were calculated using NIH Image software. (**C**) Western blot analysis of procaspase-3 in cells cultured in presence of Ac-DEVD-CHO, 24 hours after irradiation at 2 Gy. The 20 kDa band is an intermediate form of the full processing of procaspase-3. Immunodetection of caspase-3 and caspase-7 were performed with mouse monoclonal anti-CPP32 antibody and mouse monoclonal anti-MCH3 antibody respectively and Opti 4CN assay. The antibodies used do not recognize the active subunits of the respective caspases. All immunoblots were performed with equal amount of proteins in each lane

 © 2000 Cancer Research Campaign

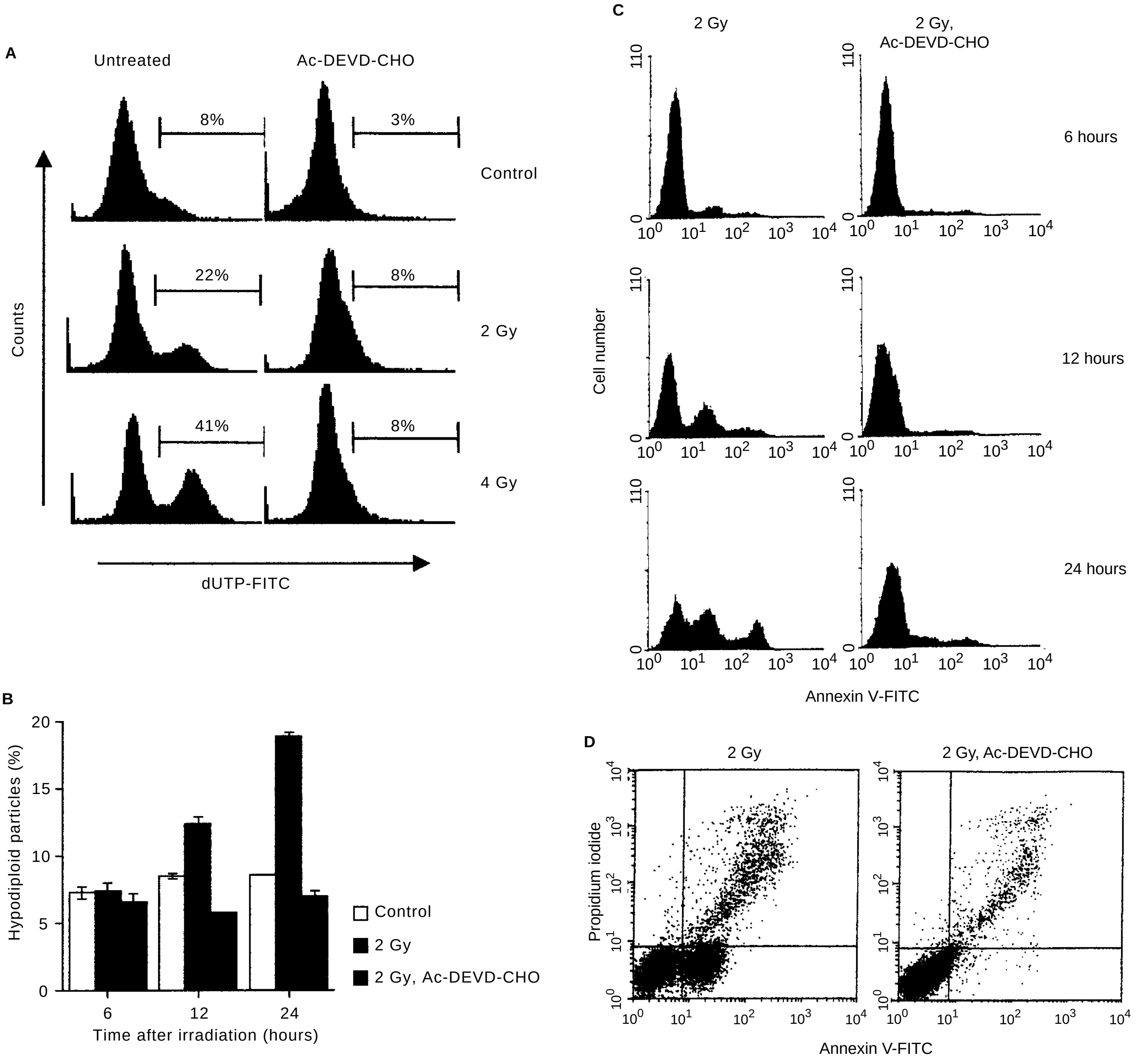

**Figure 2** Inhibition of apoptotic hallmarks by Ac-DEVD-CHO. (**A**) Incorporation of dUTP-FITC (TUNEL), measured by flow cytometry, 24 hours after X-irradiation within a range of 1 to 4 Gy. (**B**) Flow cytometry analysis quantifying hypodiploid particles with sub$G_0$–$G_1$ DNA content, excluding cellular debris, after X-irradiation at 2 Gy. At the indicated times, cells were washed, fixed and labelled with propidium iodide as described in Materials and Methods. (**C**) Kinetics of phosphatidylserine externalization with or without Ac-DEVD-CHO treatment after X-irradiation at 2 Gy. At the indicated times, cells were simultaneously stained with propidium iodide and annexine V-FITC conjugate which detect specifically exposure of phosphatidylserine on the outer leaflet of the plasma membrane and analysed by flow cytometry. (**D**) Dot-plot representation of the simultaneous staining, 12 hours after irradiation at 2 Gy. All results are representative of three independent experiments.

which is enzymatically processed into a 12 kDa subunit, a 20 kDa intermediate subunit and mature, active, 17 kDa subunit (Han et al, 1997). Although the processing of caspase-7 is less well documented, its proform (35 kDa) is also cleaved to give rise to two subunits of 11 kDa and 19 kDa (Fernandes-Alnemri et al, 1997). To define the contribution of caspase-3 *versus* caspase-7 in the proteolytic response induced by irradiation, we examined the expression of both enzymes by Western blot analysis. Western blot of actin was also performed and intensity of bands was measured with NIH Image software. The data in Figure 1B show that non-irradiated MOLT-4 cells express proforms of caspase-3 and caspase-7 but no detectable levels of active subunits. The 32 kDa procaspase-3 was down-regulated within 24 hours following an irradiation at 2 Gy, suggesting that the zymogen form was processed into its active subunits (Yu and Little, 1998). The 35 kDa proform of caspase-7 was also down-regulated following exposure to ionizing radiation in a time-dependent manner. Consistent with previous data (Han et al, 1997), the inhibition of caspase-3-like activity by Ac-DEVD-CHO treatment, prevented the appearance of the mature 17 kDa subunit and induced the accumulation of the 20 kDa intermediate (Figure 1C). Interestingly, the down-regulation of the caspase-7 zymogen seems to appear earlier

© 2000 Cancer Research Campaign than caspase-3 processing, suggesting that caspase-7 activity could act upstream of caspase-3. Thus, our results confirm that Ac-DEVD-CHO inhibits caspase-3-like activity not only by competition with endogenous substrates but also by the prevention of caspase-3 activation. These findings indicate that ionizing radiation activate both caspase-3 and caspase-7 in MOLT-4 cells.

### DNA fragmentation and hypodiploid particles induced by irradiation are prevented by Ac-DEVD-CHO

One of the most common hallmarks of the apoptotic cell is DNA fragmentation. Therefore we analysed DNA content of MOLT-4 cells by the TUNEL assay adapted to flow cytometry, 24 hours after irradiation (Figure 2A). Our results show that DNA fragmentation induced by ionizing radiation is dose-dependent and culminate in 41% of TUNEL-positive cells 24 hours after 4 Gy irradiation. Apoptosis was also assessed by propidium iodide labelling and flow cytometry analysis of irradiated MOLT-4 cells. As DNA fragmentation, hypodiploid particles formation was found to be dose-dependent following ionizing irradiation within a range of 1 to 4 Gy (Figure 2B). To determine if caspase-3-like proteases play a role in apoptotic DNA fragmentation, we pretreated MOLT-4 cells with Ac-DEVD-CHO (300 μM) 1 hour before irradiation within a range of 1 to 4 Gy. As shown in Figure 2A, inhibition of caspase-3-like proteases prevented quite completely DNA fragmentation, at both 2 and 4 Gy. The number of TUNEL-positive cells, 24 hours after irradiation at 4 Gy, decreased from 41% to 8%, and from 22% to 8% at 2 Gy, indicating that DEVD-inhibitable proteases are necessary to radiation-induced DNA fragmentation. As shown in Figure 2B, pretreatment of the cells with Ac-DEVD-CHO resulted in a marked decrease of hypodiploid particles formation, not only due to the prevention of DNA fragmentation, as assessed by the TUNEL method, but also to a slight decrease of membrane blebbing (data not shown).

### Irradiation induces phosphatidylserine externalization which is partially inhibited by Ac-DEVD-CHO

Externalization of phosphatidylserine, usually located in the inner leaflet of the plasma membrane, is one of the earliest manifestations of apoptosis, preceding DNA fragmentation and membrane blebbing (Verhoven et al, 1995). The mechanisms responsible for this asymmetry, for its preservation and for its loss remain elusive but presumably involve the activation of a non-specific scramblase and the inhibition of a specific translocase (Zwaal and Schroit, 1997). Thus, we used annexin V-FITC conjugate to detect phosphatidylserine exposure on the outer leaflet of the plasma membrane. Consistent with previous reports (Martin et al, 1996), Ac-DEVD-CHO partially prevented phosphatidylserine redistribution of the apoptotic cells (Figure 2C). The number of annexin V-positive cells, 24 hours after 2 Gy irradiation, was 64% in non-treated cells and 36% in Ac-DEVD-CHO treated cells, suggesting that the mechanisms responsible for phosphatidylserine externalization are partially sensitive to DEVDase activity, or that other molecular mechanisms are involved in apoptotic feature. Interestingly, the propidium iodide *versus* annexin V-FITC staining pattern shows an obvious difference between the cells cultured in the absence or in the presence of the caspase inhibitor (Figure 2D).

### Ac-DEVD-CHO does not prevent cell death induced by irradiation but causes necrotic death

The above mentioned data established that Ac-DEVD-CHO inhibits caspase-3-like activity and the emergence of features of apoptosis in irradiated MOLT-4 cells. Next, it was important to determine whether this compound would affect the occurrence of cell death in our model. This was evaluated using several parameters. As expected, irradiation of MOLT-4 cells caused a dose-dependent decrease in cell survival, reflected by reduced Trypan blue exclusion (Figure 3A), Alamar blue uptake (Figure 3B) and clonogenic potential. In fact, such a treatment resulted in a marked increase of necrotic death, as assessed by lactate dehydrogenase (LDH) release (Figure 3D) and by morphological criteria (data not shown). The increased release of LDH clearly demonstrates the occurrence of necrosis, since LDH release implies strong mitochondrial alteration and plasma membrane disruption. Furthermore, the simultaneous staining with propidium iodide and annexin V-FITC (Figure 2D) showed an apoptotic population in the irradiated cells (annexin V positive and propidium iodide negative) which was absent in the irradiated cells cultured with the caspase inhibitor. In this case the cells were equally positive on both stainings, suggesting that the slight increase of annexin V-positive cells was due to the loss of membrane integrity associated with necrosis rather than to the phosphatidylserine externalization associated with apoptosis. These data clearly demonstrate that co-treatment with Ac-DEVD-CHO reduced apoptosis due to ionizing radiation but did not affect the percentage of dead cells. Thus, following ionizing irradiation of MOLT-4 cells, if caspase-3-like activity is blocked, apoptosis to necrosis conversion occurs.

### DISCUSSION

In the present study, we have investigated the involvement of the caspase-3 functional subfamily in apoptosis induced by ionizing radiation and the cellular consequences of its inhibition by the reversible inhibitor Ac-DEVD-CHO (Nicholson et al, 1995; Hallan et al, 1997). This compound prevented internucleosomal DNA fragmentation and hypodiploid particle formation, indicating that the induction of such apoptotic features is dependent on caspase-3 like activity. However, Ac-DEVD-CHO inhibited only partially the externalization of phosphatidylserine, an observation consistent with earlier studies (Martin et al, 1996). In fact, it seems likely that phosphatidylserine redistribution is not due to the activity of caspase-3 itself but rather to that of another DEVD-sensitive protease since this response could be recorded in caspase-3–/– thymocytes following Fas-L ligation (Zheng et al, 1998). Here we show that MOLT-4 cells express caspase-7, an effector caspase with the same functional specificities as caspase-3 (Fernandes-Alnemri et al, 1995; Thornberry et al, 1995). Furthermore, down-regulation of procaspase-7 suggests that caspase-7 is activated following exposure of MOLT-4 cells to ionizing radiation (Figure 1B). It is therefore possible that caspase-7 represents the putative DEVD-sensitive protease distinct from caspase-3, which contributes to phosphatidylserine redistribution, both after Fas-L ligation (Martin et al, 1996) and irradiation. Further work will be required to test this hypothesis. In any case, our data are in keeping with the general notion that caspase-3-like proteases are crucially involved in most of the nuclear and cytoplasmic alterations associated with the apoptotic process (Zheng et al, 1998).

 © 2000 Cancer Research Campaign

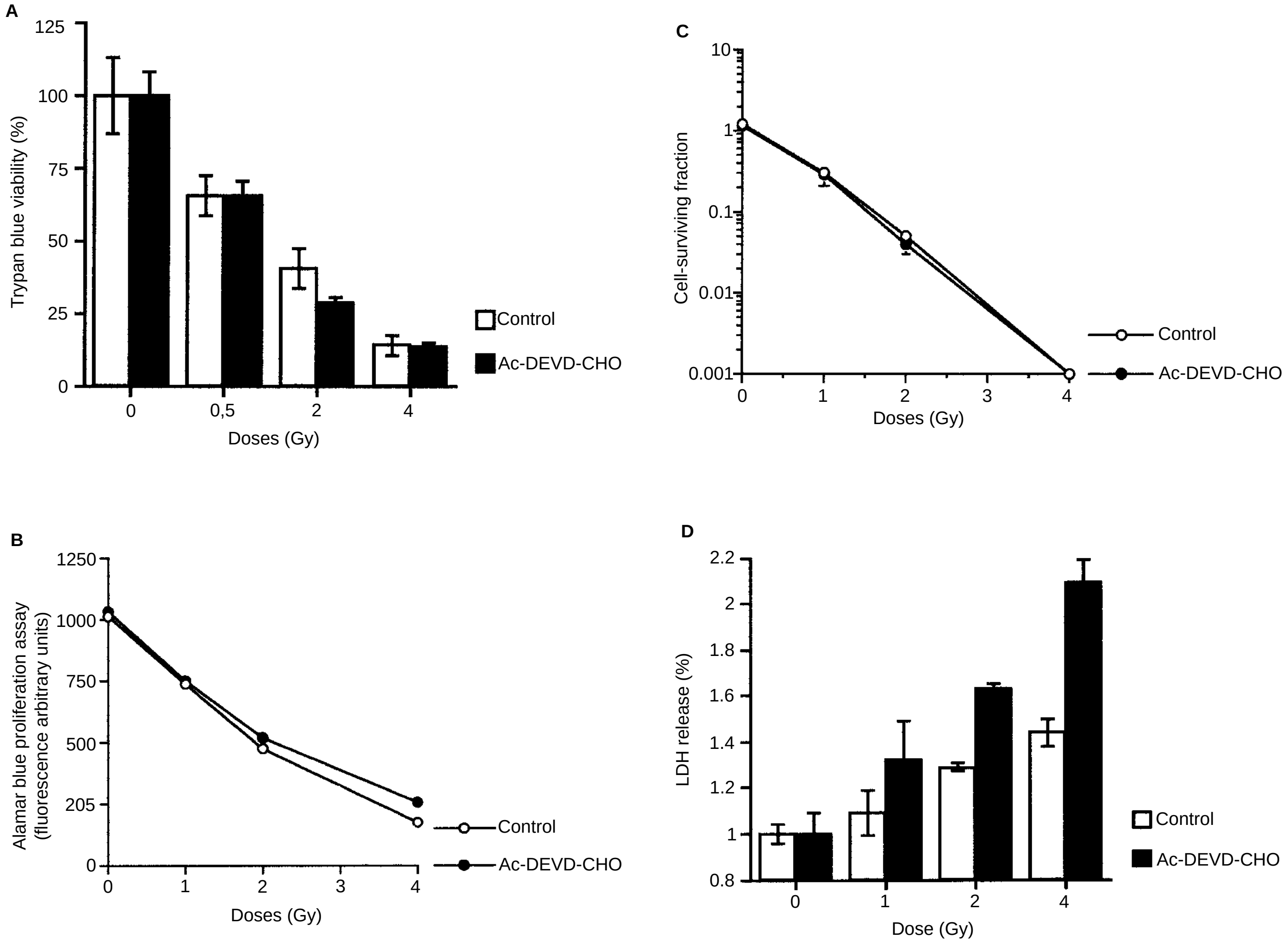

**Figure 3** Inhibition of caspase-3-like enzymes has no effect on cell viability, nor clonogenic potential and orients cell death toward necrosis. (**A**) Cell viability was determined 24 hours after X-irradiation within a range from 0.5 to 4 Gy. Reported are mean values of three independent experiments in duplicates ± SD. (**B**) Determination of cell viability by the Alamar blue proliferation assay. Alamar blue assay was performed according to the manufacturer's instructions. Briefly, 5000 cells were cultured during 4 days after X-irradiation within a range from 1 to 4 Gy. SD are, in all cases, < 10%. (**C**) Determination of the cell-surviving fraction, determined after 14 days of culture. Each point represents a mean of three independent experiments in triplicates ± SD. (**D**) The percentage of necrotic cells was determined by measuring LDH release 24 hours after X-irradiation within a range from 1 to 4 Gy in the absence and in the presence of Ac-DEVD-CHO (300 μM). Each point represents a mean of three independent experiments in duplicates ± SD.

The most important finding in the present study is that the inhibition of caspase-3-like proteases does not affect cell survival of irradiated MOLT-4 cells but leads the initiated apoptotic process towards a necrotic death. In fact, the data imply that radiation can kill by different mechanisms, depending on the enzymology of the cell. Such a switch from apoptosis to necrosis had already been described in B-lymphocytes and thymocytes treated with apoptosis-inducing drugs (Hirsch et al, 1997; Lemaire et al, 1998), in HL-60 cells treated with oxidized low density lipoproteins (Richter et al, 1996) and in camptothecin-treated U937 cells (Sané and Bertrand, 1999). Our experiments provide the first evidence that this phenomenon also occurs in the context of irradiation. Furthermore, while the previous studies utilised Z-VAD-fmk, a broad spectrum inhibitor of caspases (Hirsch et al, 1997; Lemaire et al, 1998; Sané and Bertrand, 1999), or modulation of Bcl-2 levels (Richter et al, 1996), we took advantage of the more restricted selectivity of Ac-DEVD-CHO to clearly demonstrate the role of the caspase-3 subfamily in this switch. In contrast with recent work showing that inhibition of caspases with a crmA variant, namely crmA DQMD, could enhance clonogenic survival in response to ionizing radiation in the WEHI-7 murine T lymphoblast cell line, another crmA variant, crmA DEVD, also failed to protect irradiated cells (Stefanelli et al, 1997). Thus, our results are in agreement with these data and confirm that inhibition of DEVD-sensitive caspases activity does not rescue cells from radiation-induced apoptosis but is responsible for the switch from apoptosis to necrosis. Further experiments will be required to compare the efficiency and the specificity of a DQMD-based tetrapeptide inhibitor to Ac-DEVD-CHO.

At the moment, the possible mechanism of the apoptosis to necrosis conversion recorded here and in other studies remains unclear. The formation of the apoptosome has been found to require ATP (Li et al, 1997). Therefore, ATP depletion might interfere with the initiation of the caspase cascade at this level (Eguchi et al, 1997; Leist et al, 1997). Thus, impeding the activation of effector caspases by ATP depleted conditions or inhibiting their

© 2000 Cancer Research Campaign activity with a synthetic tetrapeptide like Ac-DEVD-CHO would only make necrosis obvious, despite the prevention of apoptosis (McCarthy et al, 1997). Furthermore, it has been recently demonstrated that the failure of PARP cleavage by caspases, in fibroblasts expressing caspase-resistant PARP, could lead to the induction of necrosis (Ha and Snyder, 1999; Herceg and Wang, 1999). It will be, therefore, of interest, in future experiments, to measure intracellular ATP level in our experimental system and to assess the exact role of caspases and PARP in its regulation.

Our data may favour a model whereby apoptosis and necrosis share common initial events, presumably mitochondrial alterations, the final fate of dying cells depending on the availability of caspases acting downstream from mitochondria. We identified caspase-3-like activity as the most likely post-mitochondrial step that determines the choice between apoptosis and necrosis. The rise of DEVD-sensitive activity and the processing of caspase-3 significantly occurred only 12 hours after irradiation (Figure 1). Moreover, there was no difference in viability nor clonogenic potential whether the cells were pretreated with the caspase inhibitor 2 hours before or after the irradiation (data not shown). This suggests that caspases are not directly activated by ionizing radiation but could depend on upstream protease activity, including the mitochondrial amplification step. At least two DEVD-sensitive proteases, caspase-3 and caspase-7 seem to be activated upon exposure of MOLT-4 cells to ionizing radiation. Unlike caspase-3, active caspase-7 has been found exclusively in the mitochondrial fractions of Fas-treated hepatocytes (Chandler et al, 1998), although the full processing of procaspase-3 depends on upstream DEVDase activity (Han et al, 1997). Our results show that full processing of caspase-3 is prevented by the inhibition of DEVD-inhibitable proteases and that down-regulation of procaspase-7 takes place early after irradiation and in a time-dependent manner. Hence, it is tempting to speculate that caspase-7 might be first activated, after formation of the mitochondrial apoptosome, and would then subsequently participate in the processing of caspase-3.

The large body of experimental evidence so far accumulated has led to a fundamental distinction between apoptosis and necrosis based on the activation of caspases (Hirsch et al, 1997). Nonetheless, our study supports the recent idea that these two types of cell death are less distinctly separated than implied by the apparent morphological and functional dichotomy. Beyond considerations about the differences between an incomplete apoptosis and a necrotic death, the therapeutic potential of such a cell death conversion should be pointed out in anticancer treatment (Melcher et al, 1999; Reiter et al, 1999). Unlike chemotherapy, radiotherapy allows for a precise physical targeting of the tumour mass. The conjunction of radiotherapy with treatment by an inhibitor of caspase-3-like proteases might then be able to induce necrosis, rather than apoptosis within the tumour mass, sparing normal tissue. One might envision that necrosis and the subsequent localized inflammation would facilitate the eradication of malignant cells by enhancing the recruitment of immune cells within the tumour and by propagating damage to bystander tumour cells (McConkey, 1998; Reiter et al, 1999). Further in vivo experiments appear justified to examine the validity of this concept.

### ABBREVIATIONS

Ac-DEVD-CHO, acetyl-Asp-Glu-Val-Asp-aldehyde; Ac-DEVD-pNA, acetyl-Asp-Glu-Val-Asp-paranitroaniline; LDH, lactate dehydrogenase; PARP, poly(ADP-ribose) polymerase; CHAPS, 3-((3-cholamidopropyl)dimethylammonio)-1-propanesulphonate; DTT, dithiothreitol; EGTA, egtazic acid; PMSF, phenylmethylsulphonylfluoride; HEPES, N-(2-hydroxyethyl)piperazine-N'-(2-ethanesulphonic acid).

### ACKNOWLEDGEMENTS

We are indebted to Dr Francis Dumont (Merck, Rahway, NJ, USA) for fruitful discussions and critical review of the manuscript. This work was supported by grants from the Direction de la Recherche et de la Technologie (DRET 95–152) and Electricité de France (EDF, Service de Radioprotection).

### REFERENCES

Belka C, Marini P, Budach W, Schulze-Osthoff K, Lang F, Gilbins E and Bamberg M (1998) Radiation-induced apoptosis in human lymphocytes and lymphoma cells critically relies on the up-regulation of CD95/Fas/APO-1 ligand. *Radiat Res* **149**: 588–595

Buttke TM and Sandstrom PA (1994) Oxidative stress as a mediator of apoptosis. *Immunol Today* **15**: 7–10

Chandler JM, Cohen GM and MacFarlane M (1998) Different subcellular distribution of caspase-3 and caspase-7 following Fas-induced apoptosis in mouse liver. *J Biol Chem* **273**: 10815–10818

Cohen GM (1997) Caspases: the executioners of apoptosis. *Biochem J* **326**: 1–16

Datta R, Kojima H, Banach D, Bump NJ, Talanian RV, Alnemri ES, Weichselbaum RR, Wong WW and Kufe DW (1997) Activation of a CrmA-insensitive, p35-sensitive pathway in ionizing radiation-induced apoptosis. *J Biol Chem* **272**: 1965–1969

Eguchi Y, Shimizu S and Tsujimoto Y (1997) Intracellular ATP levels determine cell death fate by apoptosis or necrosis. *Cancer Res* **57**: 1835–1840

Fernandes-Alnemri T, Takahashi A, Armstrong R, Krebs J, Fritz L, Tomaselli KJ, Wang L, Yu Z, Croce CM, Salveson G, Earnshaw WC, Litwack G and Alnemri ES (1995) Mch3 a novel human apoptotic cyteine protease highly related to CPP32. *Cancer Res* **55**: 6045–6052

Gurtu V, Kain SR and Zhang G (1997) Fluorometric and colorimetric detection of caspase activity associated with apoptosis. *Anal Biochem* **251**: 98–102

Ha HC and Snyder SH (1999) Poly(ADP-ribose) polymerase is a mediator of necrotic cell death by ATP depletion. *Proc Natl Acad Sci USA* **96**: 13978–13982

Haimovitz-Friedman A, Kan CC, Ehleiter D, Persaud RS, McLoughlin M, Fuks Z and Kolesnick RN (1994) Ionizing radiations acts on cellular membranes to generate ceramide and initiates apoptosis. *J Exp Med* **180**: 525–535

Hallan E, Blomhoff HK, Smeland EB and Lomo J (1997) Involvement of ICE-(Caspase) family in γ-radiation-induced apoptosis of normal B lymphocytes. *Scand J Immunol* **46**: 601–608

Han Z, Hendrickson EA, Bremner TA and Wyche JH (1997) A sequential two-step mechanism for the production of the mature p17:p12 form of caspase-3 *in vitro*. *J Biol Chem* **272**: 13432–13436

Herceg Z and Wang Z-Q (1999) Failure of poly(ADP-ribose) polymerase cleavage by caspases leads to induction of necrosis and enhanced apoptosis. *Mol Cell Biol* **19**: 5124–5133

Hirsch T, Marchetti P, Susin SA, Dallaporta B, Zamzani N, Marzo I, Geuskens M and Kroemer G (1997) The apoptosis-necrosis paradox. Apoptogenic proteases activated after mitochondrial transition determine the mode of cell death. *Oncogene* **15**: 1573–1581

Leist M, Single B, Castoldi AF, Kühnle S and Nicotera P (1997) Intracellular Adenosine triphosphate concentration: a switch in the decision between apoptosis and necrosis. *J Exp Med* **185**: 1481–1486

Lemaire C, Andreau K, Souvannavong V and Adam A (1998) Inhibition of caspase activity induces a switch from apoptosis to necrosis. *FEBS Lett* **425**: 266–270

Li P, Nijhawan D, Budihardjo I, Srinivasula SM, Ahmad M, Alnemri ES and Wang X (1997) Cytochrome c and dATP-dependent formation of Apaf-1/Caspase-9 complex initiates an apoptotic protease casacade. *Cell* **91**: 479–489

Machleidt T, Geller P, Schwandner R, Scherer G and Kronke M (1998) Caspase-7-induced cleavage of kinectin in apoptotic cells. *FEBS Lett* **436**: 51–54

Martin SJ, Finucane DM, Amarante-Mendes GP, O'Brien GA and Green DR (1996) Phosphatidylserine externalization during CD95-induced apoptosis of cells and

© 2000 Cancer Research Campaign cytoplasts requires ICE/CED-3 protease activity. *J Biol Chem* **271**: 28753–28756

McCarthy NJ, Whyte MKB, Gilbert CS and Evan GI (1997) Inhibition of Ced-3/ICE related proteases does not prevent cell death induced by oncogenes, DNA damage, or the Bcl-2 homologue Bak. *J Cell Biol* **136**: 215–227

McConkey DJ (1998) Biochemical determinants of apoptosis and necrosis. *Toxicol Lett* **99**: 157–168

Melcher A, Gough M, Todryk S and Vile R (1999) Apoptosis or necrosis for tumor immunotherapy: what's in a name? *J Mol Med* **77**: 824–833

Mignon A, Rouquet N and Joulin V (1998) Les caspases, les protéases à cystéine de l'apoptose: un enjeu thérapeuthique pour demain? *Med Sci* **14**: 9–17

Nicholson DW, Ali A, Thornberry NA, Vaillancourt JP, Ding CK, Gallant M, Gareau Y, Griffin PR, Labelle M, Lazebnik YA, Munday NA, Raju SM, Smulson ME, Yamin TT, Yu VL and Miller DK (1995) Identification and inhibition of the ICE/CED-3 protease necessary for mammalian apoptosis. *Nature* **376**: 37–43

Nicoletti I, Migliorati G, Pagliacci MC, Grignani F and Riccardi C (1991) A rapid and simple method for measuring thymocytes apoptosis by propidium iodide staining and flow cytometry. *J Immunol Meth* **139**: 271–276

Reiter I, Krammer B and Schwamberger G (1999) Differential effect of apoptotic versus necrotic tumor cells on macrophage antitumor activities. *J Immunol* **163**: 1730–1732

Richter C, Schweizer M, Cossarizza A and Franceschi C (1996) Control of apoptosis by the cellular ATP level. *FEBS Lett* **378**: 107–110

Sané A-T and Bertrand R (1999) Caspase inhibition in camptothecin treated U-937 cells is coupled with a shift from apoptosis to transient G1 arrest followed by necrotic cell death. *Cancer Res* **59**: 3565–3569

Sauter B, Albert ML, Francisco L, Larsson M, Somersan S and Bhardwaj N (2000) Consequences of cell death: exposure to necrotic tumor cells, but not primary tissue cells or apoptotic cells, induces the maturation of immunostimulatory dendritic cells. *J Exp Med* **191**: 423–434

Stefanelli C, Bonavita F, Stanie I, Farruggia G, Falcieri E, Robuffo I, Pignatti C, Muscari C, Rossoni C, Guarnietri C and Caldarerra CM (1997) ATP depletion inhibits glucocorticoid-induced thymocyte apoptosis. *Biochem J* **322**: 909–917

Thornberry NA and Lazebnik Y (1998) Caspases: enemies within. *Science* **281**: 1312–1316

Thornberry NA, Rano TA, Peterson EP, Rasper DM, Timkey T, Garcia-Calvo M, Houtzager VM, Nordstrom PA, Roy S, Vaillancourt JP, Chapman KT and Nicholson DW (1997) A combinatorial approach defines specificities of members of the caspase family and granzyme B. *J Biol Chem* **272**: 17907–17911

Verhoven B, Schlegel RA and Williamson P (1995) Mechanisms of phosphatidylserine exposure, a phagocyte recognition signal on apoptotic T lymphocytes. *J Exp Med* **182**: 1597–1601

Yu Y and Little JB (1998) p53 is involved in but not required for ionizing radiation-induced caspase-3 activation and apoptosis in human lymphoblast cell lines. *Cancer Res* **58**: 4277–4281

Zheng TS, Schlosser SF, Dao T, Hingorani R, Crispe N, Boyer JL and Flavell RA (1998) Caspase-3 controls both cytoplasmic and nuclear events associated with Fas-mediated apoptosis in vivo. *Proc Natl Acad Sci USA* **95**: 13618–13623

Zhivotovski B, Joseph B and Orrenius S (1999) Tumor radiosensitivity and apoptosis. *Exp Cell Res* **248**: 10–17

Zwaal RFA and Schroit AJ (1997) Pathophysiologic implications of membrane phospholipid asymmetry in blood cells. *Blood* **89**: 1121–1132

*© 2000 Cancer Research Campaign*
